# Autofluorescence Imaging of the Skin Is an Objective Non-Invasive Technique for Diagnosing Pseudoxanthoma Elasticum

**DOI:** 10.3390/diagnostics11020260

**Published:** 2021-02-08

**Authors:** Klára Farkas, Szabolcs Bozsányi, Dóra Plázár, András Bánvölgyi, Luca Fésűs, Pálma Anker, Sára Zakariás, Ilze Lihacova, Alexey Lihachev, Marta Lange, Tamás Arányi, Norbert M. Wikonkál, Márta Medvecz, Norbert Kiss

**Affiliations:** 1 Department of Dermatology, Venereology and Dermatooncology, Semmelweis University, 1085 Budapest, Hungary; farkas.klara@phd.semmelweis.hu (K.F.); bozsanyi.szabolcs@med.semmelweis-univ.hu (S.B.); plazar.dora@phd.semmelweis.hu (D.P.); banvolgyi.andras@med.semmelweis-univ.hu (A.B.); fesus.luca@med.semmelweis-univ.hu (L.F.); anker.palma@phd.semmelweis.hu (P.A.); zakarias.sara@phd.semmelweis.hu (S.Z.); wikonkal.norbert@med.semmelweis-univ.hu (N.M.W.); medvecz.marta@med.semmelweis-univ.hu (M.M.); 2 Biophotonics Laboratory, Institute of Atomic Physics and Spectroscopy, University of Latvia, LV-1004 Riga, Latvia; ilze.lihacova@gmail.com (I.L.); aleksejs.lihacovs@gmail.com (A.L.); marta.lange.rtu@gmail.com (M.L.); 3 Department of Molecular Biology, Semmelweis University, 1085 Budapest, Hungary; aranyi.tamas@ttk.mta.hu; 4 Institute of Enzymology, Research Center for Natural Sciences, 1117 Budapest, Hungary

**Keywords:** pseudoxanthoma elasticum, autofluorescence imaging, LED, dermoscopy, quantitative analysis, calcification, diffuse reflectance imaging, diagnosis

## Abstract

Pseudoxanthoma elasticum (PXE) is a rare multisystemic autosomal recessive connective tissue disease. In most cases, skin manifestations of PXE are the first to develop, followed later by severe ocular and cardiovascular complications. In our present study, in addition to dermoscopy, we introduced novel techniques, autofluorescence (AF) and diffuse reflectance (DR) imaging for the assessment of affected skin sites of five PXE patients. PXE-affected skin areas in most skin sites showed a previously observed pattern upon dermoscopic examination. With the novel imaging, PXE-affected skin lesions displayed high AF intensity. During our measurements, significantly higher mean, minimum and maximum AF intensity values were found in areas of PXE-affected skin when compared to uninvolved skin. Conversely, images acquired with the use of 660 and 940 nm illumination showed no mentionable difference. Our results demonstrate that AF imaging may be used in the in vivo diagnostics and quantification of the severity of the skin lesions of PXE patients. In addition, it is a safe, fast and cost-effective diagnostic method. AF imaging may be also used to objectively monitor the efficacy of the possible novel therapeutic approaches of PXE in the future.

## 1. Introduction

Rare diseases may occur infrequently, yet, taken together, they affect a significant proportion of the population and place an increasing burden on society and healthcare [1]. Pseudoxanthoma elasticum (PXE, OMIM#264800) is a rare multisystemic autosomal recessive connective tissue disease. Its prevalence is estimated to be between 1 in 25,000 and 1 in 50,000, with a 2:1 female predominance [2]. PXE can be caused by over 300 mutations, located at the *ABCC6* (ATP-binding cassette subfamily C member 6) gene [3]. These mutations lead to decreased serum levels of inorganic pyrophosphate (PPi), which is an anti-mineralization factor. As a consequence, fragmentation of elastic fibers and deposition of calcium salts develop in the mid-dermis of the skin, in the Bruch’s membrane of the retina and in blood vessels [4]. Histopathological features of PXE are also characteristic for PXE-like conditions that include PXE-like syndrome with multiple coagulation factor deficiency [5] and generalized arterial calcification of infancy (GACI, OMIM#614473), which should be considered in the differential diagnosis [6]. Other dermal elastic tissue disorders, such as perforating calcific elastosis, late-onset focal dermal elastosis or papillary dermal elastolysis may clinically and histologically resemble PXE, while they mainly occur in the elderly and lack systemic manifestations [7,8]. Clinical signs of PXE usually occur during the first two decades of life. In most cases, skin lesions appear first as small, 1–5 mm yellowish papules on the neck, and similar lesions progressively develop at the periumbilical region and the flexural areas of the extremities, in particular, the axillar, popliteal and inguinal regions. Later in life, the papules coalesce into plaques and the skin becomes redundant and loose [9,10,11]. 

Ocular manifestations of PXE present most frequently with angioid streaks, that lead to choroidal neovascularization (CNV) which may associate with oedema, bleeding, chorioretinal atrophy and scars, and consequently loss of central vision [12,13]. The fragmentation of the elastic fibers and the calcification of the lamina elastica of the medium-sized arteries are behind the cardiovascular manifestations of PXE. These include premature arteriosclerosis, intermittent claudication, increased risk of early acute myocardial infarctions and stroke [14].

Dermoscopy (DS) is a widely available clinical tool, yet only a few recent studies investigated the dermoscopic features of PXE. These include yellowish-white clods, a light purplish-red background, and reticulated vessels. Yellow or white areas show differently arranged globules. The pattern of globules can present as dots, irregular broad or narrow mesh networks or parallel lines that coalesces to plaques. The vascular pattern appears as a fine reticular network or a pink background [15]. A previous study assumed that the dermal elastolysis results in the vascular realignment with a linear superficial vessel appearance [16]. Kawashima et al. described the appearance of DS images and observed the differences of dermoscopic features between PXE and PXE-like conditions. They found that the colors of the clods and the background are different, so the differential diagnosis may be possible based on DS [17]. In pilot studies, high-frequency ultrasonography (HFUS) and reflectance confocal microscopy (RCM) could detect certain characteristic features of PXE [18,19]. Ex vivo nonlinear optical microscopy was also previously introduced to visualize calcification and fragmentation of elastin fibers in PXE [20,21], among investigations on other rare disorders [22,23]. However, in vivo use of this technique was not yet reported in these indications. In a recent study, inflammation and calcification were detected in PXE-affected skin with 18-FluroDeoxyGlucose and 18F-Sodium Fluoride positron emission tomography-computed tomographic (PET-CT) imaging [24]. Limitations of this technique are the radiation exposure, low resolution and high cost.

Autofluorescence (AF) and diffuse reflectance (DR) imaging using light emitting diode (LED) illumination has been in use since the 2010s [25] and has previously been utilized to visualize different skin conditions [26,27,28]. A multispectral LED-based device prototype was successfully tested in the assessment of skin cancer and the detection of recurrence in post-operative scars [27,29]. Furthermore, it was capable to distinguish melanoma from benign lesions, including seborrheic keratosis and pigmented nevi [26]. Compared to other imaging techniques of the skin, it is a fast, cost-effective method which can be applied conveniently by any physician. The morphological and optical properties of skin lesions render PXE a strong candidate for successful use of AF imaging. In particular, the calcium salt deposits in the mid-dermis are expected to give significant AF signal based on previous spectrometric and ophthalmologic studies [30,31,32]. In our present study, in addition to DS, we set out to assess the clinical relevance of AF and DR imaging to visualize and objectify the skin lesions in PXE patients.

## 2. Materials and Methods

### 2.1. Patient Data

Three female and two male patients were included in this study, with a mean age of 55 ± 8.1 years. All patients were diagnosed and managed with PXE at Semmelweis University, Budapest, Hungary. Definitive diagnosis of the patients was established based on the revised diagnostic criteria of PXE [33], including skin biopsy and histological analysis. The diagnosis of PXE was verified by molecular genetic analysis of the *ABCC6* gene in all patients by the Center for Medical Genetics, Ghent University Hospital, Ghent, Belgium. We evaluated the severity of the disease with the Phenodex score based on clinical findings of six organ systems [34]. The patients’ data are summarized in Table 1. This study was approved by the Ethics Committee of Semmelweis University, Budapest, Hungary (SE RKEB No. 228/2018, approval date: 12 December 2018). All involved patients were informed about the study and signed the required consent.

### 2.2. Dermoscopic and Clinical Image Acquisition

Dermoscopic (Heine Delta 20, Heine Optotechnik GmbH, Herrsching, Germany) and clinical photos were collected from all PXE-affected skin sites (the neck, the periumbilical region and the flexural areas, in particular, the axillar, popliteal and inguinal regions) of the patients.

### 2.3. Autofluorescence and Diffuse Reflectance Imaging with Narrow-Band LED Excitation

A multispectral LED device prototype was used, as described earlier [35]. AF signal was excited at 405 nm, while DR images were acquired with 660 and 940 nm LEDs (SML-LXL8047UVC, Lumex Inc., Ronkonkoma, NY, USA) arranged circularly for uniform illumination with a power density of 20 mW/cm^2^. A >515 nm long pass filter was applied in order to prevent detection of 405 nm LED emission. Measurements were controlled by the direct skin contact of the device in order to block outside illumination to achieve consistent data collection. Images were collected with a color CMOS 5 megapixel IDS camera (MT9P006STC, IDS uEye UI3581LE-C-HQ, Obersulm, Germany) fixed at 60 mm distance from the illuminated skin [36]. The acquired images were automatically transferred to a cloud server for further data processing and analysis [37].

All affected skin sites of the patients were captured by this multispectral LED device. Quantitative analyses were carried out using the 405 nm AF channel and 660 and 940 nm DR images by ImageJ v1.52a software (NIH, Bethesda, MD, USA). Seven different skin sites of each patient were analyzed using the images acquired with the three different illumination wavelength settings. Altogether, 105 images were analyzed. Using the AF images, the PXE-affected areas were selected manually as regions of interest (ROI) based on their DS morphology according to patterns described earlier [15,17]. Adjacent to the affected areas, the uninvolved background skin served as the control group. Then, we transferred the selected ROI of AF images to DR images. Images were analyzed using intensity descriptors in comparison to uninvolved skin. We analyzed the mean, minimum and maximum AF intensity values within the selected ROIs. Intensity values are expressed as arbitrary units (A.U.) as measured by ImageJ software.

### 2.4. Statistical Analysis

Statistical analyses were performed with GraphPad Prism v9.0.0 software (GraphPad Software Inc., La Jolla, CA, USA) using unpaired two-tailed Student’s *t*-test. We considered *p*-values less than 0.05 statistically significant. All results are expressed as mean ± standard deviation.

## 3. Results

### 3.1. Clinical Presentation and Histopathological Features

**Figure 1:**
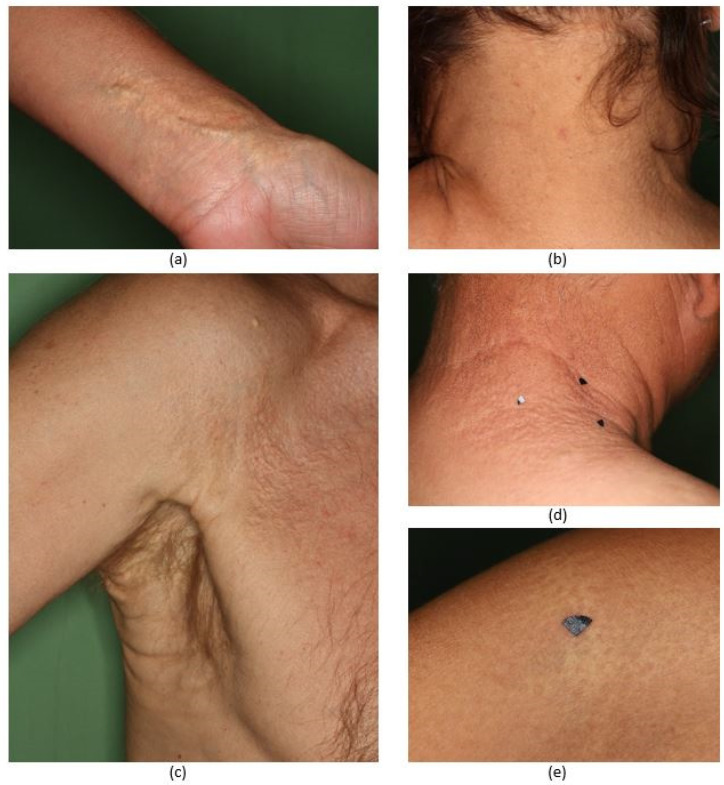
Clinical photographs that display the typical cutaneous manifestations of the patients with pseudoxanthoma elasticum. (**a**) Patient 1, large coalescent plaques on the wrist (**b**) Patient 2, 1–2 mm yellowish papules on the right side of the neck (**c**) Patient 3, redundant, loose skin in the axilla and 2–5 mm papules on the axilla (**d**) Patient 4, 2–6 mm papules on the right and dorsal side of the neck (**e**) Patient 5, right antecubital fossa, yellow-white flat plaques.

The clinical photos of the patients showed the characteristic skin changes of PXE (Figure 1). In the case of Patient 2 or Patient 5, we found moderate skin involvement, while in the rest of the cases, more extensive, prominent skin manifestations were present.

The location of the skin lesions varied between patients. Particularly, while the neck was affected in all cases, yellowish papules on the periumbilical region were only visible in two cases and the chest was affected only in Patient 1. The localization of the patients’ skin manifestations is summarized in Table 2.

**Figure 2:**
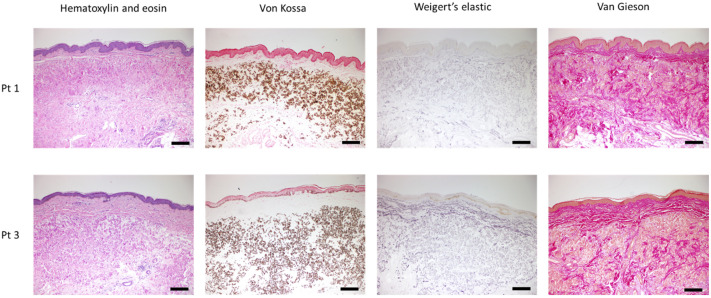
Histopathological characteristics of skin biopsies of the pseudoxanthoma elasticum patients (Pt) with hematoxylin and eosin (H&E), von Kossa (VK) staining for calcium deposits, Weigert’s elastic (WE) staining for elastin and van Gieson (VG) staining for collagen. Scale bars display 100 μm.

Skin biopsy samples were collected from clinically affected skin lesions. The histopathologic findings confirmed the diagnosis of PXE. Typical histological features were observed with specific stains by light microscopy. Hematoxylin and eosin staining showed disorganized connective tissue structure. Extensive calcium deposits were identified in the mid-dermis with Von Kossa staining. Weigert’s elastic staining revealed the fragmentation of mineralized elastin, and van Gieson staining showed abnormal, disrupted collagen fibers surrounding calcified areas (Figure 2).

### 3.2. Dermoscopy Images

**Figure 3:**
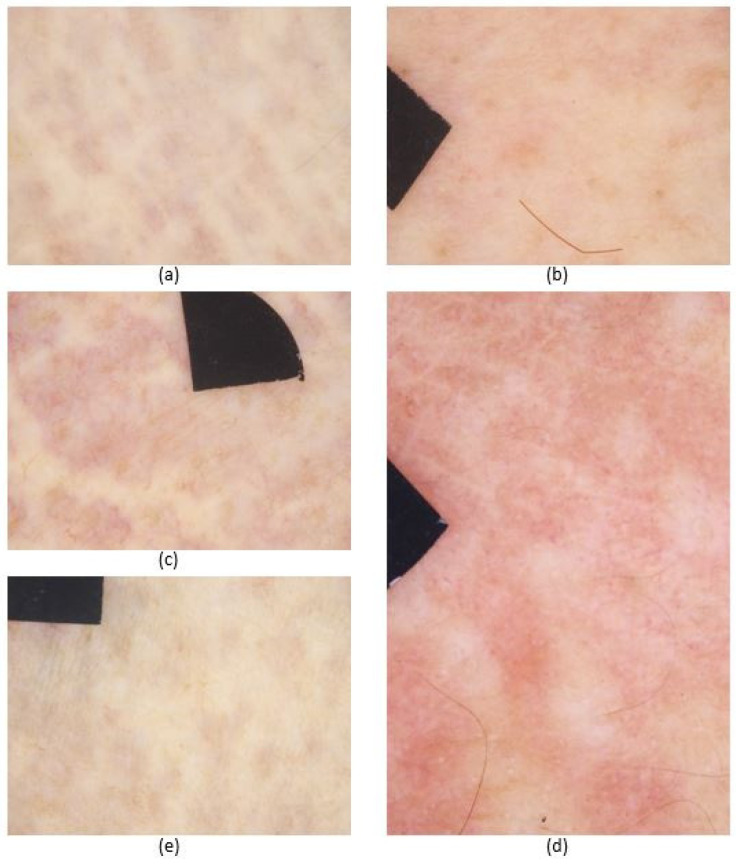
Representative dermoscopic images of the skin lesions of the patients with pseudoxanthoma elasticum. (**a**) Patient 1, linear pattern on the cubital fossa. (**b**) Patient 2, dots on the neck. (**c**) Patient 3, narrow mesh network on the axilla. (**d**) Patient 4, broad mesh network on the neck. (**e**) Patient 5, plaques of confluent dots and linear patterns in the antecubital fossa. Black markers do not point to areas of interest, they are used for image alignment (area: 0.125 cm^2^).

In the acquired DS images, in most cases, we identified yellowish clods on a light purplish-red background and reticulated vessels. The pattern of globules appeared as dots, irregular mesh networks or parallel lines (Figure 3).

However, there were some clinically affected skin sites where the specific DS features were absent (Table 3). Namely, in inguinal regions, the typical pattern appeared only in Patient 1. At the periumbilical region of Patient 1, slightly visible reticulated vessels and not obviously distinguishable globules could be detected. There were no visible DS patterns on the chest of Patient 1, the wrist of Patient 4 and the periumbilical regions of Patient 5.

### 3.3. Autofluorescence and Diffuse Reflectance Imaging

**Figure 4:**
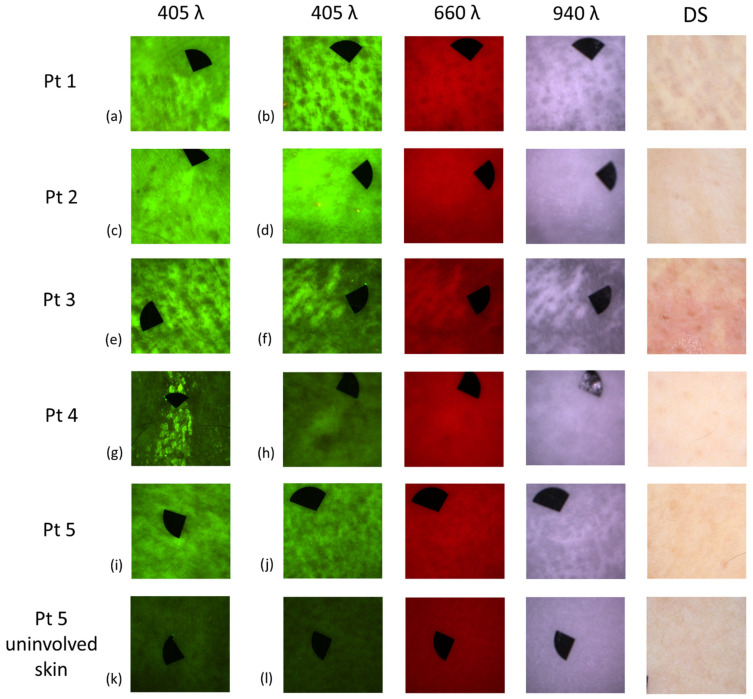
Representative autofluorescence (AF), diffuse reflectance (DR) and dermoscopy (DS) images of the affected skin sites of the pseudoxanthoma elasticum patients. (**a**) AF image of the axilla of Patient (Pt) 1. (**b**) AF, DR and DS images of the antecubital fossa of Pt 1, showing PXE-specific morphologic structures. (**c**) AF image of the axilla of Pt 2. (**d**) AF, DR and DS images of the inguinal region of Pt 2. AF image reveals a well-visible extensive area with high AF signal. DR images show barely noticeable pattern. DS image shows no typical pattern. (**e**) AF image of the antecubital fossa of Pt 3. (**f**) AF, DR and DS images of the neck of Pt 3, showing PXE-specific morphologic structures. (**g**) AF image from popliteal fossa of Pt 4. (**h**) AF, DR and DS images from wrist of Pt 4. AF image gives high-contrast signal. DR images are less informative. (**i**) AF image of the inguinal region of Pt 5. (**j**) AF, DR and DS images of the axilla of Pt 5, displaying PXE-specific morphologic structures. (**k**) AF images of Pt 5, uninvolved normal skin. (**l**) AF, DR and DS images of uninvolved normal skin, upper arm of Pt 5. The size of the images is 2 × 2 cm^2^. Black markers do not point to areas of interest, they are used for image alignment (area: 0.125 cm^2^).

The PXE-affected skin sites of each patient selected for AF and DR imaging are summarized in Table 4. AF, DR images with narrow-band LED excitation and DS findings are displayed in Figure 4. PXE-affected skin areas appeared as fields with high AF intensity with 405 nm LED excitation. With 660 nm illumination, high background skin DR signal was visible. Images acquired with 940 nm illumination showed unremarkable, low-contrast DR patterns, or in certain cases, no additional signal was detected.

AF images showed high-contrast signal of the skin lesions even in those sites of the skin where non-typical or normal DS pattern was visible. AF images acquired from the antecubital fossa and inguinal region (Figure 4d) of Patient 2 showed a well-visible affected area compared to DS images where a non-typical pattern was apparent. The AF image from the inguinal region of Patient 3 showed clearly visible lesions, but the DS image was non-typical. AF image from the wrist of Patient 4 displayed higher intensity area, while DS pattern was completely absent (Figure 4h). AF images from the inguinal (Figure 4i) and periumbilical regions of Patient 5 revealed remarkable high intensity areas, but the DS images presented non-typical or normal pattern.

**Figure 5:**
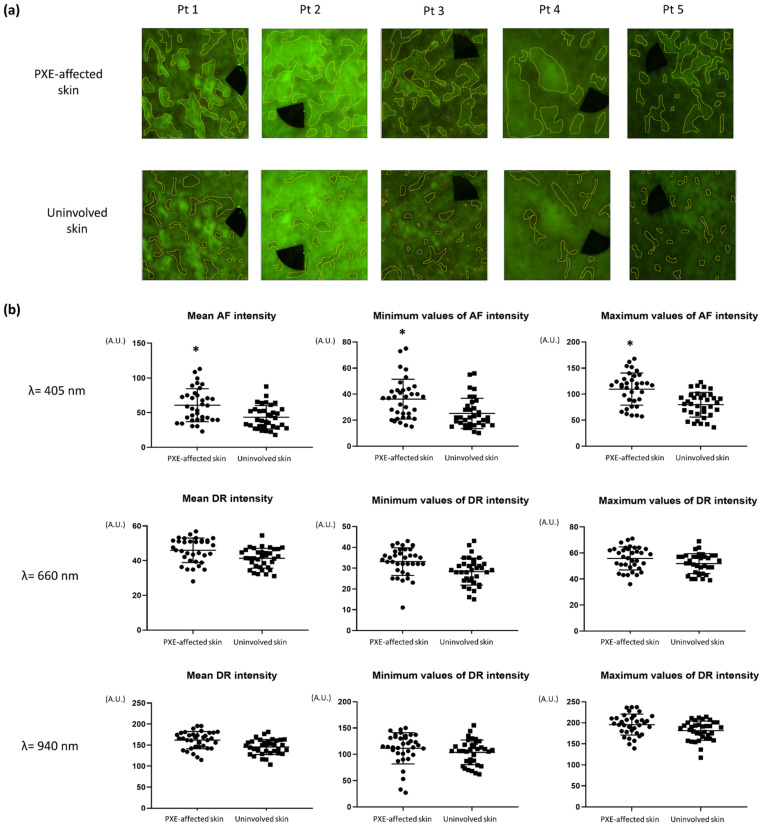
Regions of interest (ROI) selection and results of quantitative analyses of autofluorescence (AF) and diffuse reflectance (DR) images of the skin of the pseudoxanthoma elasticum (PXE) patients. (**a**) Marked PXE-affected and uninvolved skin areas as ROI. Patient (Pt) 1, neck; Pt 2, antecubital fossa; Pt 3, axilla; Pt 4, wrist; Pt 5, antecubital fossa. (**b**) In the AF images, the investigated parameters were significantly increased in PXE-affected skin compared to uninvolved skin. Analyzing DR images, no significant differences were found between PXE-affected and healthy skin. * *p* < 0.05. A.U., arbitrary unit.

Quantitative analyses revealed significantly higher mean AF intensity values in the PXE-affected skin areas compared to uninvolved skin (60.74 ± 23.86 vs. 43.40 ± 16.76 A.U.; *p* < 0.0008, Figure 5a,b). Upon measuring the minimum (36.14 ± 15.29 vs. 25.17 ± 11.66 A.U.; *p* < 0.0012) and the maximum values (109.8 ± 30.87 vs. 79.69 ± 23.76 A.U.; *p* < 0.0001) of AF intensity, we found significantly higher rates in the affected areas (Figure 5b). Analyzing DR images with 660 and 940 nm illumination of the same field of views, no significant differences were found.

## 4. Discussion

Early diagnosis of PXE is crucial to start the immediate management of the disorder which can delay or stop the progression of the systemic complications. However, to date, the diagnosis of PXE is usually delayed and often missed. The fact that most commonly the first manifestations are subtle, barely noticeable skin lesions poses a further challenge to diagnose PXE. Therefore, at the time of the diagnosis, ocular or cardiovascular complications are often already present [38].

For this reason, using a tool that enables an objective detection of the skin manifestations may open new perspectives in the diagnostic algorithm of PXE. DS is a widely available non-invasive diagnostic method, but it is difficult to notice the DS pattern of PXE, even for trained dermatologists, and requires practice. Although we also identified the previously published characteristic DS features in the PXE patients, not all affected regions show the typical patterns. Furthermore, in some PXE-affected skin sites, DS signs were completely absent. Also, DS does not allow quantification of the severity of skin involvement. The other non-invasive diagnostic modalities that may be used for the assessment of PXE also have major limitations. HFUS and RCM are both expensive techniques and require special expertise and training, and they are only scarcely available, mostly at large dermatology centers.

In our images with narrow-band LED excitation, the skin lesions of PXE patients appeared with different degrees of intensity with the different wavelengths. AF images were significantly more detailed, while DR images were generally less informative, and they did not seem to provide a significant benefit compared to dermoscopy. It was not always possible to distinguish the PXE-affected skin in DR images. In accordance, our quantitative analyses did not find significant differences between the DR images of PXE-affected skin compared to uninvolved skin.

AF imaging provides an easy and effective non-invasive diagnostic method, and probably enables the visualization of skin lesions in PXE. In skin sites which were only slightly affected by PXE, DS showed atypical pattern or DS signs were not visible. However, with the LED device, increased AF signal could still be detected. Utilizing an AF imaging device, we could markedly differentiate the PXE-affected areas from the uninvolved skin.

We suppose that the higher AF intensity of PXE-affected lesional skin could be correlated with the light absorption of calcium deposits. The peak of the absorption spectrum of ionized calcium was shown to be at 422.7 nm findings [30]. However, calcium deposits in PXE mainly consist of calcium hydrogen phosphate, calcium hydroxyapatite and, to a minor extent, iron precipitates [39,40]. Among these components, in one report, calcium hydroxyapatite was shown to harbour fluorescence properties, with a maximum excitation intensity at 405 nm [41]. However, in the paper, calcium hydroxyapatite was investigated in the form of nanoparticles. Also, it was not verified that the exact source of AF signal was calcium hydroxyapatite. As we used identical illumination wavelength of 405 nm, we hypothesise that it may be possible that the AF intensity was also emitted by calcium hydroxyapatite when present in the skin as a component of calcium deposits. In addition, calcium phosphate was reported to emit AF at 345 and 470 nm excitation wavelength using fluorescence microscopy. This could suggest that calcium phosphate should also give AF signal with 405 nm illumination [42].

Even though we could not measure the exact concentration of calcium deposits in the skin, we could recognize characteristic pattern of PXE based on the AF signal with 405 nm illumination. Moreover, DR images of the same field of views could be used to estimate relative absorption and scattering properties of the examined skin lesions. As seen, the DR images showed high reflection in the exact same areas that showed high AF signal. According to the optical properties of the skin (including fluorophores and chromophores), absorbers and scatters have minimal absorption and scattering in the red and near-infrared (NIR) region [43,44]. In the DR channels of PXE-affected skin, we could detect increased reflection which presumably is not related to the skin major chromophores and fluorophores, as they have a minimum absorption and scattering in the red and NIR region. By comparing the images (AF and DR channels), we could clearly see that PXE lesions produce high AF intensity signal with 405 nm excitation, as well as demonstrate high optical reflection visible in DR images with 660 and 940 nm illumination. Analyzing the histopathological images, the structures responsible for high AF signal should be calcium deposits. However, the origin of high AF and reflection signal could be attributed to main tissue compounds present among calcium deposits visualized by histology. The mineralization of elastin fibers and alterations of the collagen structure are also histopathological features of PXE [21]. Therefore, in addition to calcium compounds, elastin and collagen as endogenous fluorophores could also be responsible for the increased AF signal [45]. Additional studies would be necessary to clarify the exact origin of high AF signal in PXE lesions under 405 nm excitation. Also, identification of the optimal LED illumination wavelength for AF could further increase the selectivity of the imaging for skin lesions of PXE.

In this work, in addition to DS, we performed quantitative AF imaging and successfully distinguished PXE-affected skin from uninvolved skin areas using intensity descriptors with the aim to objectify the degree of skin involvement. A recent retrospective clinical study of 125 PXE patients found a significant correlation between the number of the PXE-affected skin sites of and the occurrence of severe cardiovascular events and/or ophthalmological complications [46]. In addition, in a smaller cohort of 14 patients, similar results were reported regarding the cardiovascular involvement [47]. These studies indicate that the accurate assessment of the skin manifestations could also indicate the degree of systemic involvement in PXE and could harbor great prognostic value in the future.

Here, we introduced a LED-based fluorescence imaging device as a potential non-invasive diagnostic tool for the assessment of the skin of PXE patients. Our results demonstrate that AF imaging is capable of the in vivo detection of PXE-affected skin and the quantification of the severity of the skin lesions of PXE patients. Moreover, it is a safe, fast and cost-effective diagnostic approach. AF imaging may be also used to objectively monitor the efficacy of the possible novel therapeutic approaches of PXE in the future.

## Figures and Tables

**Table 1:** Demographic data and Phenodex score of the PXE patients.

Pt. No.	Sex	Age (y)	Phenodex Score
1	F	53	S3 E3 G0 V1 C0 R0
2	F	53	S2 E3 G0 V1 C1 R0
3	M	69	S3 E3 G0 V0 C0 R0
4	M	52	S2 E3 G0 V0 C1 R0
5	F	48	S2 E2 G0 V0 C0 R0

Pt, patient; F, female; M, male; y, years; S, skin; E, eye; G, gastrointestinal; V, vascular; C, cardiac; R, renal; PXE, pseudoxanthoma elasticum.

**Table 2:** Affected skin sites of the pseudoxanthoma elasticum patients.

	Pt 1	Pt 2	Pt 3	Pt 4	Pt 5
neck	+	+	+	+	+
axilla	+	(+)	+	+	(+)
antecubital fossa	+	(+)	+	(+)	+
popliteal fossa	+	(+)	+	(+)	(+)
inguinal	+	+	+		(+)
periumbilical	+				+
wrist	+			(+)	
chest	(+)				

Pt, patient; +, clearly visible papules or plaques; (+) mild, poorly visible skin lesions.

**Table 3:** Characteristic dermoscopic features of the affected skin sites of the pseudoxanthoma elasticum patients according to the nomenclature of Berthin et al. [15] and Kawashima et al. [17].

	Structure	Pt 1	Pt 2	Pt 3	Pt 4	Pt 5
neck	globules	plaques	dots, linear	mesh network	linear	dots, plaques
	background	red	brownish	purple-red	red	brownish
	vessels	reticulated, linear	reticulated	reticulated	reticulated	no
axilla	globules	plaques	dots	mesh network	dots	dots
	background	purple	brownish	purple-red	pink	brownish
	vessels	linear	no	reticulated	no	no
antecubital fossa	globules	linear	(non-typical)	linear	linear	plaque
	background	purple-red		purple	brownish	purple
	vessels	reticulated		reticulated	no	no
popliteal fossa	globules	linear	linear	linear	dots	linear
	background	purple-red	brownish	purple	pink	purple
	vessels	several reticulated	no	reticulated	reticulated	reticulated
inguinal	globules	dots	(non-typical)	(non-typical)		(non-typical)
	background	purple				
	vessels	reticulated				
periumbilical	globules	dots				(absent)
	background	purple-red				
	vessels	reticulated				
wrist	globules	plaques			(absent)	
	background	purple				
	vessels	reticulated				
chest	globules	(absent)				
	background					
	vessels					

Pt, patient. Blank cells indicate that DS images were not captured in that region, due to the lack of cutaneous manifestations.

**Table 4:** The investigated skin sites of the pseudoxanthoma elasticum patients.

	Pt 1	Pt 2	Pt 3	Pt 4	Pt 5
Site 1	popliteal fossa	popliteal fossa	popliteal fossa	popliteal fossa	periumbilical
Site 2	Inguinal	inguinal	inguinal	axilla	inguinal
Site 3	periumbilical	axilla	inguinal	axilla	axilla
Site 4	axilla	axilla	axilla	axilla	antecubital fossa
Site 5	wrist	antecubital fossa	antecubital fossa	wrist	antecubital fossa
Site 6	antecubital fossa	neck	neck	antecubital fossa	neck
Site 7	neck	neck	neck	neck	neck

## Data Availability

The data presented in this study are available on request from the corresponding author. The data are not publicly available due to ethical considerations.
